# Metabolic Impact of Frailty Changes Diabetes Trajectory

**DOI:** 10.3390/metabo13020295

**Published:** 2023-02-16

**Authors:** Alan J. Sinclair, Ahmed H. Abdelhafiz

**Affiliations:** 1 Foundation for Diabetes Research in Older People (fDROP), King’s College, London WC2R 2LS, UK; 2 Department of Geriatric Medicine Rotherham General Hospital, Rotherham S60 2UD, UK

**Keywords:** older people, diabetes mellitus, frailty, management, hypoglycaemic therapy

## Abstract

Diabetes mellitus prevalence increases with increasing age. In older people with diabetes, frailty is a newly emerging and significant complication. Frailty induces body composition changes that influence the metabolic state and affect diabetes trajectory. Frailty appears to have a wide metabolic spectrum, which can present with an anorexic malnourished phenotype and a sarcopenic obese phenotype. The sarcopenic obese phenotype individuals have significant loss of muscle mass and increased visceral fat. This phenotype is characterised by increased insulin resistance and a synergistic increase in the cardiovascular risk more than that induced by obesity or sarcopenia alone. Therefore, in this phenotype, the trajectory of diabetes is accelerated, which needs further intensification of hypoglycaemic therapy and a focus on cardiovascular risk reduction. Anorexic malnourished individuals have significant weight loss and reduced insulin resistance. In this phenotype, the trajectory of diabetes is decelerated, which needs deintensification of hypoglycaemic therapy and a focus on symptom control and quality of life. In the sarcopenic obese phenotype, the early use of sodium-glucose transporter-2 inhibitors and glucagon-like peptide-1 receptor agonists is reasonable due to their weight loss and cardio–renal protection properties. In the malnourished anorexic phenotype, the early use of long-acting insulin analogues is reasonable due to their weight gain and anabolic properties, regimen simplicity and the convenience of once-daily administration.

## 1. Introduction

The global diabetes prevalence is about 10.5% of adults aged 20–79 years, and this is expected to rise by 46% in the year 2045 [1]. Almost half of the population with diabetes is above the age of 65 years due to increased life expectancy in this age group, and the prevalence is highest (24%) at the of age 75–79 years [1]. In older people with diabetes, frailty emerges as an additional complication to the traditional micro- and macrovascular disease. This is due to the increasing prevalence of diabetes-related complications and diabetes-associated comorbidities in old age [2,3]. Frailty is defined as a state of increased vulnerability to psychological and physical stressors because of a reduction in physiological reserve, which limits the capacity to maintain homeostasis [4]. The prevalence of frailty increases with increasing age, reaching around 25% in people aged ≥85 years and affects about 32–48% of older people with diabetes [4,5]. The prevalence of frailty is generally higher in older people with diabetes compared to those without diabetes [6]. Frailty is associated with adverse outcomes, including increased risk of disability, hospitalisation and mortality [7,8,9,10,11]. In addition to the adverse outcomes, frailty may induce body composition and metabolic changes, which may have an impact on the trajectory of diabetes, and changes the phenotypes of older people with diabetes [12]. Current guidelines view older people categorically as frail or non-frail with no consideration of metabolic changes induced by frailty and its effect on the therapeutic options or the glycaemic targets [13,14]. Therefore, this manuscript reviews the metabolic changes associated with frailty, its impact on diabetes trajectory and suggests therapeutic options and glycaemic goals in frail older people with diabetes based on their metabolic phenotypes.

## 2. Diabetes and Frailty

**Figure 1:**
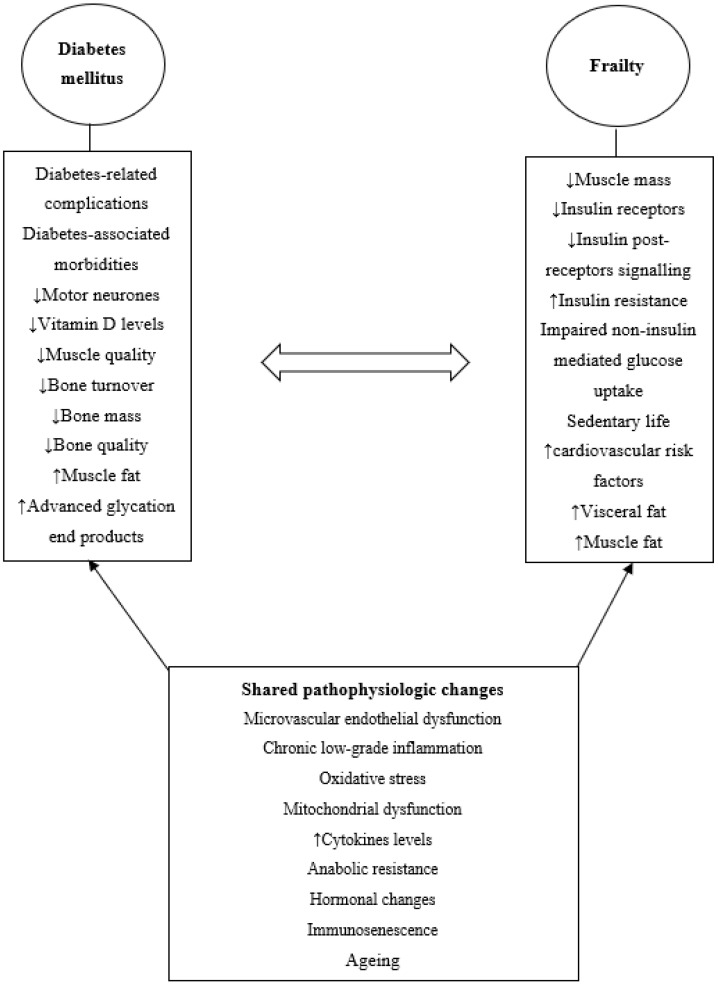
Bidirectional relationship between diabetes mellitus and frailty.

Diabetes and frailty are two linked conditions, and the relationship appears to be bidirectional. Hyperglycaemia increases the risk of frailty even in individuals with pre-diabetes [6]. Several studies shown that the risk of frailty increases proportionally with hyperglycaemia and HbA1c levels [15,16,17,18]. The association between diabetes and frailty is incremental when diabetes-associated comorbidities, such as hypertension, and diabetes-related complications, such as peripheral neuropathy, are present [19,20]. Hypoglycaemia increases the risk of incident frailty by 44% [21]. Hypoglycaemia also increases the risk of fractures, which may eventually lead to frailty [22,23,24]. Similarly, low blood glucose or low HbA1c is associated with an increased risk of frailty and disability [25,26,27]. Therefore, glycaemia and frailty appear to have a U-shaped relationship [28]. It is likely that hyperglycaemia increases the risk of frailty due to the associated risk of micro- and macrovascular complications. In addition, hyperglycaemia-related low-grade chronic inflammation, oxidative stress and mitochondrial dysfunction are other factors increasing the risk of frailty [29,30,31]. On the other hand, hypoglycaemia may increase the risk of frailty through the wide variations in blood glucose levels, which may lead to endothelial damage and vascular disease [32]. Low HbA1c is associated with other conditions such as cognitive decline and cerebrovascular disease, which may contribute to the development of frailty [33,34]. For example, although low HbA1c was associated with disability and care-need, in one study, dementia was the main driver for this need [27]. In a bidirectional relationship, frailty and pre-frailty have been shown to increase the risk of incident type 2 diabetes mellitus [35]. This could be because frailty is associated with glucose dysregulation and insulin resistance [36] (Figure 1).

## 3. Diabetogenic Effects of Ageing

Ageing is associated with body composition changes that increase the risk of diabetes [37]. For example, with increasing age, muscle mass declines, which is the major tissue responsible for glucose uptake, and visceral fat increases leading to increased insulin resistance and glucose intolerance [38]. Age-related muscle loss is progressive with ageing by about 6% per decade starting from midlife, which proportionally increases insulin resistance as we get older [39]. In addition, the reduction of energy consumption due to less physical activity with increasing age leads to overall weight gain, which is largely due to fat accumulation rather than lean muscle mass gain [40]. Another contributing factor is the change in fat distribution with ageing, which includes loss of subcutaneous fat and an increase in visceral fat as well as the deposition of ectopic fat, especially intra-muscularly, which further increases insulin resistance [41]. Moreover, there is a reduction of the β-cells number and function with increasing age, reducing insulin secretion by about 0.7% per year [42]. Ageing is associated with physiologic disturbance in glucagon-like peptide-1 (GLP-1) and gastric inhibitory polypeptide (GIP), which have a role in maintaining β-cells growth and proliferation, contributing to β-cells dysfunction [43,44]. Reduced sex hormone secretion with ageing results in a reduction of lean muscle mass, increased visceral fat, reduction of subcutaneous fat and weight gain, which is another contributing factor to increased insulin resistance with ageing [45,46,47,48]. This age-related muscle–fat distribution leads to a reduction in the secretion of the beneficial myokines by the muscle cells and increased secretion of the harmful adipokines by the fat cells, resulting in an imbalance that promotes an unfavourable metabolic state such as increased insulin resistance, oxidative stress, low-grade inflammation, mitochondrial dysfunction and risk of diabetes [49,50]. With increasing age and associated visceral obesity, early reversible microvascular endothelial dysfunction occurs, which progresses over time leading to established diabetes-related vascular complications [51]. 

## 4. Diabetes Trajectory

Environmental factors, in addition to genetic variants, are associated with the development of type 2 diabetes [52]. Obesity and insulin resistance are the two major factors that predispose to glucose intolerance and diabetes [53]. Insulin resistance develops because of the deposition of ectopic fat in skeletal muscles, the liver and pancreas, which may also lead to reduced β-cells function and number [54]. Type 2 diabetes develops when β-cells fail to secrete a compensatory amount of insulin to overcome the insulin resistance. An increase in insulin secretion initially compensates for hyperglycaemia to maintain blood glucose levels in the normal range [55]. Over time, insulin hypersecretion will not be enough to control the blood glucose levels in the normal range, which goes through a continuum of pre-diabetes then to overt diabetes. Pre-diabetes (impaired fasting glucose and/or impaired glucose tolerance) are conditions where blood glucose levels are higher than normal but not yet reaching the diabetic range [56]. With the ongoing decline in β-cells, pre-diabetes progresses, and hyperglycaemia becomes persistent then overt diabetes develops. Therefore, the natural trajectory of diabetes is progressive with persistent hyperglycaemia, which will not respond to a change in lifestyle and diet alone but will need treatment initially with oral and then eventually injectable therapy to maintain the blood glucose levels in the normal range [56]. 

## 5. Impact of Frailty on Body Composition

Frailty is associated with muscle mass loss or sarcopenia, and muscle weakness is a criterion of frailty [2]. However, frailty does not affect the loss of different muscle fibres equally. With the development of frailty, there is a significant loss of type II more than type I muscle fibres [57]. Type II fibres have less oxidative but more glycolytic properties than type I fibres. Due to the low oxidative properties of type II fibres, they tend to have more intramuscular lipid storage increasing insulin resistance and glucose intolerance [58]. On the contrary, type I fibres, due to their high oxidative qualities, have less muscular fat storage and higher insulin sensitivity. Therefore, overall individual insulin sensitivity is affected by which muscle fibre type is dominant. A muscle biopsy study showed a significant loss of type II muscle fibres and a severe reduction in the type II/I size ratio in frail older women, with a mean age (SD) of 77.9 (6.2) [59]. Another study showed a significant reduction of type II compared to type I (47% vs. 17%) in pre-frail men [60]. Increasing age is also associated with significant type II muscle fibres atrophy, which accounts for most of the muscle loss in old age [61,62]. 

Therefore, frailty, overall, is associated with the accelerated loss of predominantly type II muscle fibres that may reduce insulin resistance [12]. 

## 6. Impact of Frailty on Diabetes Trajectory

**Figure 2:**
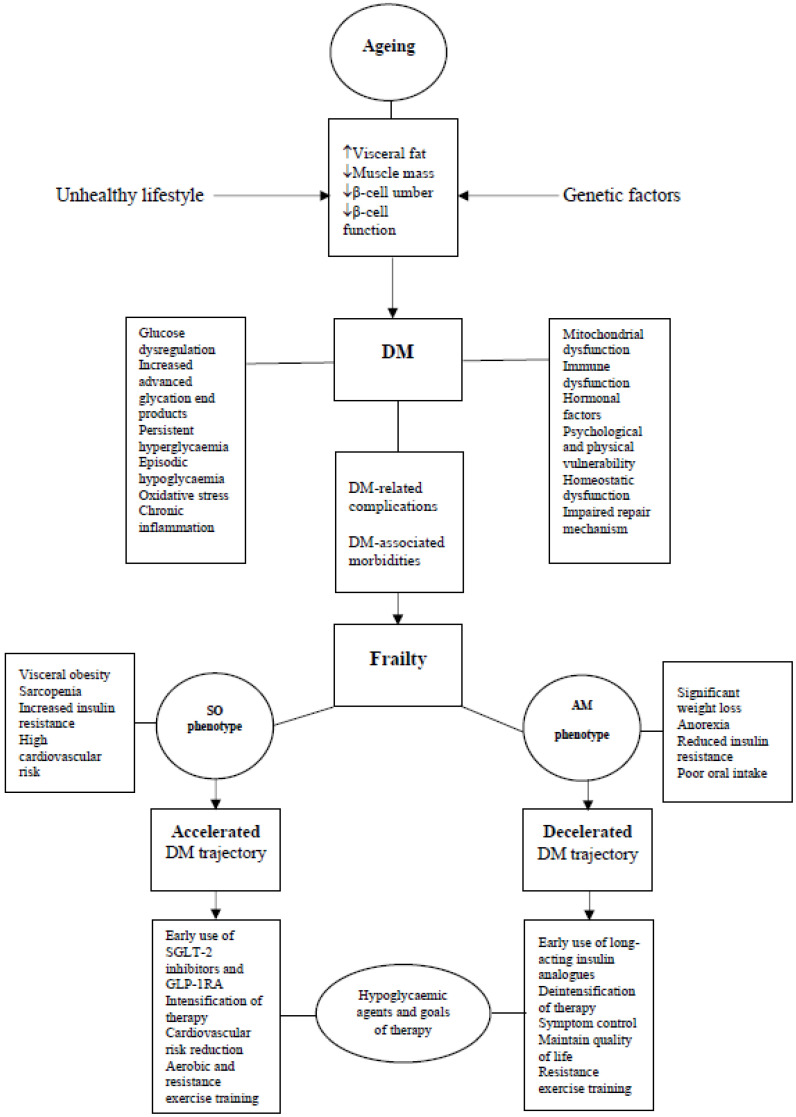
Ageing, diabetes and frailty. Age-related body composition changes predispose to glucose intolerance. In the presence of environmental and genetic factors, diabetes develops. Diabetes-related complications and associated comorbidities lead to emergence of frailty. Frailty metabolic phenotypes affect diabetes trajectory. Diabetes trajectory is accelerated in SO phenotype, which requires intensification of therapy, while it is decelerated in AM phenotype, which requires deintensification of therapy. DM = diabetes mellitus, SO = sarcopenic obese, AM = anorexic malnourished.

Frailty is perceived as a vulnerability to stressors due to reduced physiologic reserves, but the full understanding of frailty and its associated metabolic changes is still unclear [63]. The development of frailty affects body composition in a way that changes the metabolic profile, and this will have an impact on diabetes trajectory. In addition to frailty-associated weight loss, frailty leads to muscle mass loss or sarcopenia, which is a condition closely linked to frailty [64,65]. Furthermore, a frail individual can also suffer from obesity rather than weight loss, which is not a mandatory requisite for frailty diagnosis [64]. The relationship between frailty and body mass index (BMI) has been shown to be U-shaped [66]. Therefore, with the differential loss of muscle fibres, muscle mass, visceral fat accumulation and weight loss, the overall insulin sensitivity will vary from one frail person to another. Frailty can be seen as a metabolic disease ranging from an anorexic malnourished individual at one end to a sarcopenic obese individual at the other end as part of a metabolic spectrum. In between these two phenotypes, there will be individuals with varying degrees of fat/muscle ratios and corresponding varying degrees of insulin sensitivities. It is likely that the above-mentioned two phenotypes at both ends of the spectrum have a significant metabolic impact on diabetes trajectory (Figure 2). 

### 6.1. Sarcopenic Obese Phenotype (SO)-Accelerated Diabetes Trajectory

In the sarcopenic obese phenotype, diabetes trajectory is accelerated due to the increase in visceral fat and the decrease in muscle mass, which both increase insulin resistance and promote persistent hyperglycaemia. In addition, the sarcopenic obese individuals will have an overall unfavourable metabolic profile and dyslipidaemia [67]. There is also differential loss of the oxidative type I muscle fibres relative to the glycolytic type II fibres in a sarcopenic obese person, which further favours insulin resistance and unfavourable metabolism [68]. Furthermore, the sedentary life style in obese sarcopenic individuals increases intramuscular fat deposition, which further increases insulin resistance. The development of sarcopenic obesity exaggerates age-related muscle–fat imbalance. Skeletal muscle is an endocrine organ which secretes mediators known as myokines. The myokines promote a favourable metabolism such as increasing insulin sensitivity, glucose uptake, fatty acid oxidation and energy expenditure [69,70]. Similarly, adipose tissue exhibits endocrine function by secreting adipokines, which are important mediators of various metabolic processes such as fatty acid oxidation. However, in obesity, adipose tissue becomes dysfunctional, promoting a pro-inflammatory, hyperlipidaemic and insulin-resistant state [71]. Therefore, in sarcopenic obesity, the age-related muscle–fat imbalance is accelerated, promoting an unfavourable metabolic state that further increases the risk of diabetes-associated cardiovascular disease by increasing oxidative stress and mitochondrial dysfunction [49]. 

### 6.2. Anorexic Malnourished Phenotype (AM)-Decelerated Diabetes Trajectory

In the anorexic malnourished phenotype, diabetes trajectory is decelerated due to significant weigh loss. Anorexia and reduced protein intake increase with increasing age in some older individuals [72,73]. This is particularly common in individuals living in care homes [74]. Anorexia can be physiologic due to age-related changes in the gastrointestinal system such as impaired chewing, oesophageal reduced motility, reduced salivary and gastric secretions, and this can be exacerbated by pathologic factors due to comorbidities, especially dementia and frailty [75,76]. The prevalence of anorexia appears to be similar to frailty. The general prevalence of anorexia is around 21.2% and is highest (34.1%) in care homes residents [74]. Similarly, the prevalence of frailty was 12.7 to 28.2% in one study and was also highest (19.5 to 44.1%) in care home settings [77,78]. In the AM phenotype, there is a predominant loss of the insulin-resistant type II muscle fibres, which leads to reduced insulin resistance. One study showed increased insulin resistance in frail older people only when abdominal obesity was present, while insulin resistance was the same in non-obese frail compared with healthy older persons [79]. Weight loss is known to improve insulin sensitivity in internal organs such as the liver and skeletal muscles and reduce fat deposition in the pancreas, which may also improve insulin secretion from the β-cells [80,81]. Therefore, some patients with diabetes in this phenotype have shown spontaneous resolution of hyperglycaemia and normalisation of HbA1c [82]. 

## 7. Clinical Implications

Due to the heterogeneity, frailty should not be seen as a single category of patients. Therefore, the clinical practice of the choice of hypoglycaemic agents and setting goals of therapy should be precisely tailored according to the frailty metabolic phenotype. For example, in the SO frail phenotype, the new therapies of sodium-glucose transporter-2 (SGLT-2) inhibitors and the glucagon-like peptide-1 receptor agonists (GLP-1RA) are a reasonable priority, while in the AM frail phenotype, early use of insulin should be considered. 

## 8. SO Phenotype

### 8.1. Hypoglycaemic Agents—Early Use of SGLT-2 Inhibitors and GLP-1RA

The SO frail phenotype patients are likely to gain most from the new therapies of SGLT-2 inhibitors and GLP-1RA due to its favourable metabolic and weight-losing properties. Compared to obesity or sarcopenia alone, the sarcopenic obesity is associated with an unfavourable metabolic state of insulin resistance, persistent hyperglycaemia, dyslipidaemia, hypertension and metabolic syndrome, which are associated with an increased risk of cardiovascular disease and major cardiovascular adverse events, including mortality [83,84,85,86]. 

In addition, obesity increases the risk of chronic kidney disease (CKD) and albuminuria [87]. The mechanisms include increased secretion of pro-inflammatory adipokines by fat cells and increased insulin resistance, which affect glomerular function and increase albuminuria [88]. Furthermore, obesity is associated with non-alcoholic fatty liver disease (NAFLD), which is a spectrum of liver diseases associated with the adverse metabolic conditions of insulin resistance, dysglycaemia and hypertension and is associated with increased cardiovascular risk [89]. The new therapies have significant cardio–renal properties, which are essentially required in the SO phenotype patients [90]. GLP-1RA and SGLT-2 inhibitors showed a significant reduction in major adverse cardiovascular events, cardiac and total mortality, stroke, non-fatal myocardial infarction and hospitalisation for heart failure [91,92,93,94]. In addition, SGLT-2 inhibitors have shown a beneficial effect of microvascular endothelial function in animal studies, which may have a positive scope in humans [95]. SGLT-2 inhibitors are associated with significant reductions in albuminuria and a reduced rate of composite renal endpoint (progression to macroalbuminuria, doubling of serum creatinine, progression of estimated glomerular filtration rate (eGFR) to ≤45 mL/min/1.73 m^2^, initiation of renal replacement therapy or death from renal disease) [96,97]. SGLT-2 inhibitors are associated with an average 2–4 kg reduction in body weight by promoting daily urinary excretion of 60–80 g of glucose and a loss of 240–320 calories, and this osmotic diuresis also reduces blood pressure by 5–6/1–2 mmHg [98]. Consistent with the efficacy of the new therapies in the overall population, the cardio–renal protection has been demonstrated in older people (≥65 years), as recently reported by a meta-analysis of clinical trials [99]. Both GLP-1RA and SGLT-2 inhibitors improve liver enzymes in patients with non-alcoholic fatty liver disease (NAFLD) and histological features of non-alcoholic steatohepatitis (NASH) and reduce liver fat content [100,101,102]. The new therapies improve liver pathology through their properties of reducing total body fat, lipogenesis, inflammatory markers and oxidative stress and stimulating the oxidation of free fatty acids [103,104]. In addition to the favourable metabolic profile of these new therapies in SO patients, they also have advantages for frailty. For example, the intervention arms in the randomised clinical trials have consistently demonstrated a low risk of hypoglycaemia, which is comparable to, or even lower, than that of the placebo arms [105]. This means that hypoglycaemic therapy can be intensified to achieve target glycaemic control with less risk of hypoglycaemia, which is higher in frail compared to non-frail, older people with diabetes [106]. Another advantage is the reduction of polypharmacy, which is associated with negative consequences such as medication side effects and non-compliance. The SO individuals are likely to have associated comorbidities of the metabolic syndrome, and the use of the new therapies may reduce the burden of other medications, such as diuretics and antihypertensives, in these patients [105]. 

### 8.2. Goals of Therapy-Intensification Approach

Due to the unfavourable metabolic state in the SO phenotype, intensification of hypoglycaemic therapy with a focus on cardiovascular risk reduction is required. Clinical guidelines are focused on function and recommend tighter targets in functionally independent and relaxed goals in those with poor function [107,108,109]. However, SO frail phenotype patients are likely to benefit from the new therapy based on their metabolic profile rather than their function [110]. In addition, the cardio–renal protective effects occur early in treatment and are not related to HbA1c levels, suggesting that this frail phenotype will still benefit, even if they do not have a long life expectancy [105]. There is no specific glycaemic target for each metabolic phenotype, as this has not yet been investigated in the literature. A general target of HbA1c < 7.5–9.0% (<59–75 mmol/mol) is suggested by the guidelines for general frail older people [104,105,106]. However, a lower target of HbA1c < 7.0% (53 mmol/mol) may be associated with better physical function [111]. In addition to pharmacologic therapy of cardiovascular risk factors such as anti-hypertensive, cholesterol lowering and antiplatelet therapies, improving muscle power may reduce cardiovascular risk [112]. Exercise training combined with adequate nutrition may help restore muscle mass and function [113]. Muscle contraction during exercise training releases protective myokines, which reduce the harmful effects of the pro-inflammatory adipokines associated with sarcopenic obesity [114]. Resistance exercise training increases muscle mass, while aerobic exercise reduces body fat, therefore a combination of both types of exercise is superior to either of them alone [115]. Other forms of exercise, such as aquatic exercise, may be useful in patients with arthritis [115]. Whole-body electromyostimulation, yoga, vibration or tai chi are other forms of exercise for people with disabilities [116,117]. Adequate nutrition management is required to reduce body fat and reach an ideal body weight without muscle mass loss. A diet with a daily protein intake of 1–1.2 g/Kg, which includes essential amino acids, especially leucine, is needed to maintain muscle mass [118,119]. 

## 9. AM Phenotype

### 9.1. Hypoglycaemic Agents—Early Use of Insulin

Due to significant weight loss and malnutrition in this frailty phenotype, insulin appears to be a useful early use choice due to its anabolic properties. In addition, this frailty phenotype is likely to be more prevalent in an advanced age population with poor oral intake, who are intolerant to oral therapy due to multiple comorbidities and less compliance with medications. The anabolic properties of insulin include the control of peripheral tissues’ glucose uptake, glycogenesis, lipogenesis and skeletal muscle protein synthesis resulting in weight gain [120,121]. It also reduces the net glucose production by suppressing glycogenolysis and glycolysis [122]. Insulin facilitates protein synthesis through the control of the amount of branched-chain amino acids taken by the skeletal muscles [123]. The anabolic effects of insulin may extend to other organs’ functions, such as promoting bone formation and attenuating osteoporosis-related inflammation [124]. Previous studies suggested that the anabolic effects of insulin are blunted with increasing age, and higher doses may be required in older people [125,126]. However, more recent studies demonstrated that endogenous insulin is significantly and positively correlated with skeletal muscles mass; low insulin levels are associated with sarcopenia, and insulin therapy improved the skeletal muscle index and muscle mass and delayed the progression of sarcopenia [127,128,129,130]. The main disadvantage of insulin therapy is the parenteral route of administration and the risk of hypoglycaemia. Therefore, the decision of choosing which type of insulin depends on the level of the associated risk of hypoglycaemia, the inconvenience of the injection frequency, accessibility and cost [131]. Long-acting insulin analogues are suitable in this frail phenotype due to their convenient once-daily administration and low risk of hypoglycaemia due to their less prominent peak levels and long duration of action. Recent studies have demonstrated superior efficacy and safety of insulin analogues compared to intermediate-acting human insulins [132,133,134,135]. Insulin analogues have been shown to reduce glucose variability, improve glucose control, enhance quality of life, reduce emergency department visits or hospital admission due to severe hypoglycaemia and reduce the overall reduce risk of hypoglycaemia, especially nocturnally, compared with intermediate-acting human insulins [132,133,134,135]. The simplicity and safety of insulin analogue regimens may overcome the traditional barriers of insulin initiation such as anxiety about frequent injections, fear of hypoglycaemia, complex regimens, blood glucose monitoring and support burdens [136,137]. 

### 9.2. Goal of Therapy—Deintensification Approach

Due to the significant weight loss and anorexia in this frailty phenotype, they are likely to need less and less hypoglycaemic therapy. In addition, the risk of hypoglycaemia in this phenotype with possible erratic oral intake is likely to be high [103]. Therefore, a deintensification of therapy approach is appropriate. Hypoglycaemic therapy has been successfully deintensified or completely withdrawn in frail older people with diabetes without worsening HbA1c levels [138,139]. The characteristics of these patients were care home residency, multiple comorbidities, old age, low HbA1c and significant weight loss [138,139]. With the wide range of glycaemic targets suggested by the clinical guidelines for HbA1c < 7.5–9.0% (<59–75 mmol/mol), the higher end of HbA1c (8.0–8.9%, 63.9–73.8 mmol/mol) was associated with better outcomes in frail patients who needed skilled assistance or fulfilled criteria for institutionalisation [111]. However, a higher HbA1c > 9.0% (75 mmol/mol) was associated with increased mortality [140]. These patients are likely to have multiple comorbidities, which is a potential competitor for the benefit of tight glycaemic control, further suggesting that relaxed targets are appropriate in this population [141]. In addition to long-term targets, due to short life expectancy, a focus on the short-term targets is important. For example, keeping random blood glucose levels between 6–15 mmol/L is important to avoid hypo- and hyperglycaemia, reduce malaise, improve mental function and maintain the quality of life [142,143]. Long-acting insulin analogues will be an appropriate early choice of therapy in this anorexic malnourished frail phenotype as long as hypoglycaemia is avoided [144]. Hypoglycaemia should be clearly avoided in this frailty phenotype, as it is associated with an increasing risk of falls and disability [145]. In addition to pharmacologic therapy, adequate nutrition as possible and resistance, rather than aerobic, exercise training may help improve function. A multimodal intervention of individualised and progressive resistance exercise training for 16 weeks and a structured diabetes and nutritional educational programme over seven sessions, along with optimal diabetes care, resulted in clinically relevant and cost-effective improvements in the functional status of frail older people with diabetes [146]. 

## 10. Conclusions

In old age, frailty is an emerging new complication that is closely linked to diabetes mellitus. Frailty appears to have a wide spectrum of metabolic phenotypes, which impacts on diabetes trajectory. The SO phenotype is characterised by enhanced insulin resistance due to a combination of increased visceral fat and reduced skeletal muscle mass, which accelerates diabetes trajectory. On the other hand, the AM frailty phenotype is characterised by significant weight loss and reduced insulin resistance, which lead to deceleration of diabetes trajectory. The new therapies of SGLT-2 inhibitors and GLP-1RA are a reasonable early choice of therapy in the SO frail phenotype due to their weight loss and cardioprotective properties. The focus is intensification of therapy and cardiovascular risk reduction. Long-acting insulin analogues, on the other hand, are more suitable early choices in the AM frail phenotype due to their anabolic and weight gain properties, ease of administration and lower risk of hypoglycaemia compared with intermediate-acting human insulins. The focus is deintensification of therapy, symptom control and maintenance of the quality of life. 

## 11. Future Perspectives

We have suggested that the two ends of the frailty spectrum, the SO and AM, are likely to have significant metabolic differences that affect diabetes trajectory. Full exploration of the metabolic spectrum of frailty still needs future exploration. However, studies reporting an association of low glycaemia with frailty demonstrated common features of participants, which included lower body weight and malnutrition compared with non-frail patients, suggesting an AM phenotype [147]. On the other hand, other studies reporting an association of high glycaemia with frailty demonstrated common features of participants, which included higher body weight, higher waist circumference and higher cholesterol compared to non-frail patients, suggesting a SO phenotype [148,149,150,151]. The association of frailty with both low and high glycaemia can be explained by the presence of two metabolic rather than one phenotype of frailty, which needs future confirmation [152]. The suggestion that diabetes may have different phenotypes with different insulin resistance and disease trajectory has been previously reported [153]. Further investigation in this area is required to accurately sub-stratify patients with diabetes and guide precision medical practice. It is important in future clinical trials to stratify the phenotypes of frail patients from the outset to precisely identify which hypoglycaemic agent and what glycaemic target is suitable in each phenotype. The promising extra glycaemic effects of SGLT-2 inhibitors and GLP-1RA may reduce the development of cardio–renal complications, which may have a positive impact on the rate of incident frailty. In addition, GLP-1RA has been shown, in animal studies, to improve cognitive function, reduce cerebral accumulation of amyloid-b peptide in Alzheimer’s disease, influence dopamine levels in Parkinson’s disease and demonstrated beneficial effects on brain ischaemia. Therefore, there is the potential of these agents to maintain neuronal integrity and improve neurodegenerative diseases in humans [154]. The effect of these new therapies on skeletal muscles and muscle function needs future investigation. Although some studies showed increased incidence of falls in patients taking GLP-1RA compared to other hypoglycaemic agents and the effect of SGL-2 inhibitors on sarcopenia is inconsistent, other studies suggested that SGLT-2 inhibitor therapy is associated with an improved muscle quality, and the net effect on muscle is likely beneficial [155,156,157,158,159,160]. Similarly, studies are required to further investigate the potential anabolic effect of insulin on muscle function in frail older people with diabetes. The recent suggestion of a once-weekly insulin analogue is promising and is a more convenient regimen for frail older people, which may further encourage the early use of insulin in these vulnerable frail patients [161]. 

## 12. Key Points

Frailty is a common diabetes-related complication in older people.

Frailty is likely to have a wide metabolic spectrum, which ranges from a sarcopenic obese phenotype at one end and an anorexic malnourished phenotype at the other end.

The sarcopenic obese phenotype is associated with accelerated diabetes trajectory and needs therapy intensification.

The anorexic malnourished phenotype is associated with decelerated diabetes trajectory and needs therapy deintensification.

Early use of SGLT-2 inhibitors and GLP-1RA in the sarcopenic obese and long-acting insulin analogues in the anorexic malnourished phenotype is appropriate.
